# Annotations

**Published:** 1897-11-27

**Authors:** 


### ANNOTATIONS.

### Once for All.

The report of the Howard Association contains, among other interesting matter, a protest against the practice of recommitting a prisoner who has just come out of prison for another offence than the one for which he has been punished. The irritation of such a proceeding is indisputable; and it is enough to make a criminal who has formed some good resolutions during his imprisonment fall back into his evil courses again. If there are two offences to be dealt with, let them be punished at the same time. Indeed, one might wish a criminal worse than that he should have all his offences run into one big crime, and punished by a long term of imprisonment. Short sentences do nothing except to add to the apparent number of our criminals. If Jane Cakebread comes up for the hundredth time to receive sentence for drunkenness, what does it do but add to the number of her convictions? In a few days she will be out again, thirsting for liquor, which, when she has obtained it, will send her back to the court, to be imprisoned once more. Jane and those who resemble her must spend months of their life in prison every year, but does any one think that they are thereby likely to be reformed? If all the terms were run into one, there might be some chance; but a few days' imprisonment to a drunkard means only that he will come out mad for drink; a few days' imprisonment to a thief means only that he is furious with himself for having bungled his work; a few days' imprisonment to one who has been guilty of assault is to send him out determined to punish the person who has brought punishment upon him. With a longer term of incarceration good influences might have a chance to work; there might be some chance of repentance, and at least the prisoner might have learned some work by which he could earn his bread honestly.

### The Cost of Strikes.

At the present moment, when the public eye is turned to a great labour contest, it may be worth while to consider what profit the engineers would get if they succeeded in their aim. We all know what suffering a strike causes while it lasts; we hear of the loss to trade, and we do not need to be told that if a strike is unsuccessful it is a long time before the worker regains his former position. But strikes sometimes succeed, and then there is great rejoicing among the working class. The masters have been conquered, and it is true that the masters suffer; but does that imply that the workmen have gained? The best answer to this will probably be given by quoting some statistics which are given by Professor Walker in his book on "Wages." He says that, assuming the point at issue to be a rise of five per cent. in wages, the result will be as follows, even supposing that the strike succeeds: The loss of one lunar month's wages will require to make up for it work for one year and three-fifths, even at the higher rate of pay. Two months' idleness will demand extra work for three and one-fifth years before the money lost can be made up. A six months' strike means that it will take nine years and three-fifths to make up the loss; and if the strike goes on for twelve months and a half, not less than twenty years of work will be needed to compensate the workman. Of course, this does not inevitably mean that strikes are always foolish and wrong. If the working class are willing to suffer for the sake of gaining a profit, which many of them will never live to get the whole benefit of, good and well. It is thus only that great victories are won, and they may be content to think that those who come after them will reap the benefit; but if their agitation is purely for their own benefit it is well worth their while to ask themselves if they are really going to gain anything; and above all, it is worth while to make sure, before they strike, that if they gain the day it will be a permanent advantage to them.

### The Wounded in Battle.

In the presence of so many little wars, and with the possibility of large ones hanging constantly over our heads, it is a matter of no small importance to consider what is to happen to the enormous number of wounded who will assuredly bestrew the great battle-fields of the future. The rapidity with which modern repeating rifles and machine guns can be fired, and their great length of range, will bring upon any contested spot a perfect hail of bullets, and unless battles are to be won purely by strategy the losses must be enormous. What, then, is to be done with the wounded? Experiments made in the German army show very clearly that, unless we were prepared very largely to increase the number of stretcher-bearers, it would be quite impossible, even as a mere physical problem, to carry the wounded off the field. But it would also be impossible in consequence of the risks to which the bearers would be exposed, which would be far greater than those of ordinary combatants—so great, in fact, that according to many competent authorities the whole organisation would be absolutely annihilated. Archibald Forbes has given it as his opinion that the service, as now existing, would be found utterly impracticable in the new conditions, and that the first battle would bodily wipe out the bearer organisation carried on under fire. Bardeleben also shows that to increase the number of bearers is but to increase the number of wounded, and thus to require the devotion of a still larger number to the service of removal; and he also concludes that the whole system of removing the wounded on litters during the battle must be abandoned. If this is so, our efforts for the relief of the wounded will have to be concentrated chiefly on two points—first, in giving immediate succour where they lie; and, secondly, on rapid removal as soon as firing ceases. So far as immediate succour on the field is concerned, it is clear that it not only will be more than ever necessary that the medical officers should accompany the troops into the fighting line, but that the troops themselves must be thoroughly well drilled in the application of first aid. For this purpose it is not enough that they should be trained as bearers. If the wounded are to receive that help on the field which is absolutely necessary in many cases, if they are to weather the period which must elapse before they can be removed to a place of safety, their brother soldiers must be definitely taught exactly what to do in the more common emergencies of the battle field. The rest must be dealt with by the medical officer on the spot.

